# Relationship between knowledge, adherence-related behaviors and self-management with response to monoclonal antibody therapy in patients with severe asthma

**DOI:** 10.1038/s41598-026-64363-5

**Published:** 2026-07-31

**Authors:** Christopher Alexander Hinze, Vincent Müller, Christina Valtin, Frederik Trinkmann, Katrin Milger, Nora Drick, Hendrik Suhling, Jan Fuge

**Affiliations:** 1 Department of Respiratory Medicine and Infectious Diseases, Hannover Medical School, Carl-Neuberg-Straße 1, Hannover, 30625 Germany; 2 German Center for Lung Research (DZL), Thoraxklinik Heidelberg, Translational Lung Research Center Heidelberg (TLRC), University Hospital Heidelberg, 69126 Heidelberg, Germany; 3 Department of Medicine V, Ludwig-Maximilians-University of Munich (LMU), University Hospital, Munich, Germany; 4 Biomedical Research in Endstage and Obstructive Lung Disease Hannover (BREATH), German Center for Lung Research (DZL), Hannover, Germany

**Keywords:** Severe asthma, Severe eosinophilic asthma, Biologics, Monoclonal antibody therapy, Treatment response, Medication adherence, Adherence-related behavior, Patient activation (PAM), Asthma knowledge, Health literacy, Diseases, Health care, Immunology, Medical research

## Abstract

This study explores the relationship between patient activation, adherence behaviors, asthma knowledge, and response to monoclonal antibody (mAb) therapy in severe eosinophilic asthma (SEA). Despite advances in mAb therapies, some SEA patients continue to experience uncontrolled symptoms. Understanding these factors could lead to more effective, personalized treatment strategies. Participants diagnosed with SEA and undergoing mAb therapy for 6-9 months were recruited from three German university outpatient clinics. They were categorized using the Biologics Asthma Response Score (BARS) into responders, partial responders, and non-responders. Data collection focused on demographic details, lung function, medication history, patient activation (PAM13-D), and adherence (A14 questionnaire). Statistical analyses assessed the impact of adherence and knowledge on treatment outcomes. Of the 140 participants, 61% were responders, 24% partial responders, and 15% non-responders. Age, sex distribution, smoking status, pack-years, eosinophil levels, ExNO and lung function parameters were comparable across response groups. A14 scores and asthma knowledge differed significantly across response groups, whereas PAM13-D did not. In ordinal logistic regression, asthma knowledge remained associated with BARS-defined response category after adjustment. A negative attitude towards drugs was associated with response category in univariate analysis, but this association was attenuated after adjustment. Better asthma knowledge was associated with BARS-defined response to mAb therapy, while PAM13-D and overall A14 score were not independently associated with response. Medication attitude may still be clinically relevant, but their association with response should be interpreted cautiously. These findings support a patient-centered approach that considers asthma knowledge, adherence-related behaviors and medication attitudes during biologic treatment. Prospective studies are needed to determine whether targeted education and adherence support can improve clinical outcomes.

**Supplementary Information:**

The online version contains supplementary material available at 10.1038/s41598-026-64363-5.

## Introduction

Asthma bronchiale is a widespread lung disease characterized by airway inflammation, varying respiratory symptoms and variable expiratory airflow limitation with a profound impact on patients’ well-being and healthcare resources. Despite strides in asthma management, a subset of individuals suffer from severe eosinophilic asthma (SEA) in consequence of frequent exacerbations, insufficient disease control and obstructive airways under highest-dosed inhaler therapy^1^. In recent years, monoclonal antibody (mAb) therapies have emerged as an effective frontier in SA treatment^2–5^. These innovative therapies target specific inflammatory pathways implicated in asthma pathogenesis, offering a tailored approach to disease management. By zeroing pivotal components of the immune response, such as interleukins and immunoglobulins, monoclonal antibodies hold the potential to improve airway inflammation, mitigate exacerbation rates and enhance lung function in patients suffering from SEA.

Although several monoclonal antibodies have been approved for use in severe asthma, there remains a subset of patients who do not achieve full therapeutic benefit from these biologic agents^6^. Despite their targeted mechanism of action and demonstrated efficacy in some patients, a significant proportion continue to grapple with uncontrolled symptoms and persistent exacerbations, underscoring the heterogeneous nature of SEA and the need for further therapeutic innovation^7^.

Patient adherence to asthma treatment remains a challenge due to various factors including cost and complexity^8^. Non-adherence to inhaler medications leads to uncontrolled symptoms and exacerbations, posing risks to patients and burdening healthcare systems^9^. Concerning mAb-therapy, race, administration setting and access to specialist care have been identified as decisive factors for adherence^10–12^ whereas adherence to antibodies was observed to be higher than to inhaler therapy^13^. Participation, motivation, adapted behaviors and caring for one’s own illness, summarized as the patient specific activation and self-management plays a pivotal role, especially in the case of chronic illnesses. It has been shown that activation is high in patients with asthma, but a non-negligible proportion exhibit low activation. This had a negative impact on disease control and could be improved by specific intervention^14,15^. Yet, to our knowledge, it has not been shown how patient activation impacting therapy response in patients with SEA undergoing mAb therapy.

Disease management programs (DMP) have been part of non-medicinal asthma therapy for many years and aim to improve disease control through training and knowledge transfer. Results from several studies from different countries have revealed potential benefit of patient education to improve disease specific outcome parameters^16–19^. In recent decades, several questionnaires have been developed to assess patient-specific asthma knowledge^20,21^.

This study aims to elucidate the interplay between patient activation, adherence-related behaviors, asthma knowledge and response to monoclonal antibody treatment in a large cohort of patients receiving mAb-therapy for severe asthma. The primary endpoint is the association of patient activation measured by PAM13-D, adherence-related behaviors measured by the A14 questionnaire, and asthma knowledge measured by KISS with BARS-defined treatment response after 6–9 months of mAb therapy. Secondary endpoints included associations between BARS-defined treatment response and A14 subdomains, PAM13-D activation levels, adherence category, and patient-reported quality of life. By investigating these relationships, the study seeks to shed light on patient-related factors associated with treatment outcomes, and to support the development of more effective and tailored therapeutic strategy for this patient population.

## Material and methods

### Participants

Patients diagnosed with SEA and mAb therapy for at least 6 to 9 months were recruited for the study from three German university outpatient clinics for severe asthma between November 2022 and November 2023: Ludwig-Maximilian-Universität in Munich, Thoraxklinik Heidelberg and Medizinische Hochschule Hannover. Severe eosinophilic asthma was diagnosed by the treating severe asthma specialists according to current guideline-based criteria, including severe asthma requiring high-dose inhaled corticosteroid therapy and evidence of type 2/eosinophilic inflammation, defined as a blood eosinophil count of ≥300 cells/µL before initiation of mAb therapy. Patients were categorized into three groups based on treatment response using the Biologics Asthma Response Score (BARS) as a rapid tool to reliably assess response to mAb therapy: responders, partial responders and non-responders^22^. The BARS rates the Asthma Control Test (ACT)^23^, the annualized exacerbations and dosage of corticosteroids between 0 and 2 points each. The rounded mean value will lead to the BARS (0 points = non-responder; 1 point = partial-responder and 2 points = responder). Non-German speaking patients were excluded as proper understanding of the questionnaire cannot be ensured. Only complete questionnaires with an answer for every question were considered for analysis.

### Data collection

In this cross-sectional study, clinical data were collected from participants, including demographic information, clinical characteristics, medication history and psychosocial factors. Demographic data included age, gender, and smoking status. Clinical characteristics encompassed lung function parameters as well as biomarkers such as eosinophil levels and exhaled nitric oxide (ExNO) at the time point of study inclusion. Medication history focused on the type of monoclonal antibody therapy received. Psychosocial factors included participation, motivation and adapted behaviors measured by the Patient Activation Measure 13 (PAM13-D)^24,25^, adherence-related behaviors assessed by the A14 questionnaire^26^, and quality of life evaluated using a visual analogue scale (VAS) between 0 and 10 points. The PAM13-D score ranges from 0 to 100 points while a cutoff at 47, 55.1 and 72.5 points discriminates between level 1, 2, 3 and level 4 patient activation, respectively. It is a validated tool available in German language and provides insight into a patient’s readiness and ability to engage in self-care. The A14-questionnaire is a validated tool for use in clinical routine, available in German language without the need for a license. It has been developed to assess generic medication use and scores from 0 to 56 points, where 50 points (90%) and above are classified as adherent. The A14 consists of four subscores: non-intentional non-adherence due to forgetfulness; patients’ own regimen adaptations due to safety or efficacy reasons; non-adherence due to practical obstacles in daily life such as cost and expenditure of time of the therapy and non-adherence due to a negative attitude towards drugs. In this study, the A14-questionnaire reflects general medication-use behaviors (including inhaled medication). Asthma Knowledge was assessed with 55 questions from the patient-completed asthma knowledge questionnaire^21^ using right- or wrong answers. Domains of asthma knowledge comprised therapy, triggers, symptoms, asthma control, medication, mAb and consequences of uncontrolled asthma. We adapted the questionnaire and additionally included six true/false items addressing key aspects of monoclonal antibody therapy (identification of biologics, approximate annual costs, continuation of inhaled therapy, residual exacerbation risk, dosing interval/route, and the need for ongoing outpatient follow-up) and named it the **K**nowledge and act**I**vation in a**S**thma **S**urvey, KISS. The additional biologic-specific items were developed by severe asthma specialists to capture practical knowledge considered relevant for patients receiving mAb therapy; however, these added items were not formally validated as an independent questionnaire module. Each right answer was rated with 1 point resulting in a summarized score between 0 and 55 points, with a higher score indicating higher asthma knowledge. Supplementary questions (without impact on the asthma knowledge score) asked about patients’ enrollment in asthma management programs, participation in asthma training, history of lung rehabilitation, interest in further information about asthma and preferred methods of receiving additional information. Comorbidities, asthma duration, polypharmacy, previous mAb use, mAb switching history, educational level, and socioeconomic variables were not systematically captured in a standardized manner across all study sites and were therefore not included in the analyses.

### Data analysis

IBM SPSS Statistics version 29 and R-Studio using R 4.3.3 were used to analyze the data. Statistical analysis was conducted to assess differences in patient activation, adherence-related behaviors, asthma knowledge and other variables across responder, partial responder, and non-responder groups. Descriptive statistics such as median and interquartile range (IQR) or mean and standard deviation (SD) were used to summarize continuous variables, while categorical variables were shown using frequencies and percentages. Group differences were analyzed using appropriate statistical tests, including Kruskal-Wallis test for continuous variables and chi-square test for categorical variables. Only complete questionnaires with an answer for every item were included in the respective questionnaire analyses. Missing clinical data were not imputed. Regression analyses were performed as available case analyses using complete observations for the variables included in each model. The primary endpoints were analyzed by simple and multiple ordinal logistic regression to evaluate the associations of A14 score, A14 subdomains, PAM13-D score and KISS on non-response category of BARS. To reduce model instability and avoid combining conceptually related questionnaire domains, PAM13-D, A14 and KISS were analyzed in separate models. Adjusted models included predefined covariates available at study inclusion, including age, sex, smoking status, eosinophil levels and tezepelumab therapy. The A14 subdomains were analyzed in the same way as exploratory secondary analyses. Biologic therapy distribution was analyzed descriptively, because treatment allocation was not randomized and subgroup sizes differed between biologics, no formal comparative efficacy analysis between individual biologics was performed. The proportional odds assumption was assessed using the Brant test. No adjustment for multiple comparisons was performed, and p-values should therefore be interpreted as exploratory. Significance was set at p < 0.05.

### Ethical considerations

The study was conducted in accordance with ethical guidelines and approved by the institutional review board of Hannover Medical School Number (10624_BO_K_2022 and Nr. 2923-2015). Informed consent was obtained from all participants prior to their inclusion in the study.

## Results

### Patient characteristics and antibody treatment

**Fig. 1:**
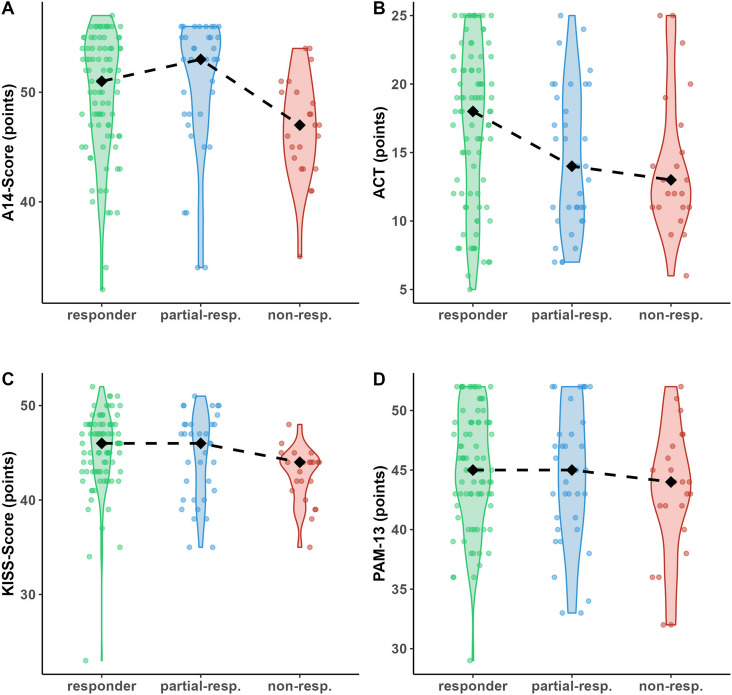
Comparison between responder, partial-responder and non-responder for (**A**) A14, Adherence to Treatment Questionnaire, (**B**) Asthma-Control Test (ACT), (**C**) Asthma Knowledge Score (KISS, The **K**nowledge and act**i**vation in a**s**thma **s**urvey), (**D**) Patient Activation Measure (PAM13-Score). Data are presented as violin plots with Median and individual data points.

The study encompassed 140 participants, categorized into responder (n=86, 61%), partial responder (n=33, 24%) and non-responder (n=21, 15%) groups. Age and sex distribution were comparable across response groups, with no statistically significant differences between responders, partial responders, and non-responders (female sex: 58%, 52%, and 43%, respectively, p=0.423; age: 59 [50–65], 55 [51–62], and 57 [51–63] years, respectively, p=0.607). Smoking status and pack-years also did not differ significantly across response groups (former smokers: 40%, 58% and 43%, respectively, p=0.205; pack-years: 10 [2.8–24.8], 20 [5–30] and 10 [9–20], respectively, p=0.596). Further analysis revealed no statistically significant disparities in eosinophil levels, ExNO or lung function parameters across response groups (Table 1, Figure 1). Tezepelumab was used significantly more often in partial-responder and non-responder (<0.001, Table 1). The association between tezepelumab therapy and response category was statistically significant (Fisher’s exact test p<0.001), with a Cramer’s V of 0.294, indicating a small-to-moderate association.

**Table 1:** Patient demographics and clinical data.

	**All** **n=140**	**Responder** **n=86 (61%)**	**Partial responder** **n=33 (24%)**	**Non-responder** **n=21 (15%)**	**p-value**
*Female sex – n (%)*	76 (54)	50 (58)	17 (52)	9 (43)	0.423
*Age – Median (IQR)*	57 (51; 64)	59 (50; 65)	55 (51; 62)	57 (51; 63)	0.607
*BMI – Median (IQR)*	28 (24; 32)	27 (24; 32)	28 (24; 30)	29 (24; 35)	0.646
***mAb-Therapy – n (%)***					
*- Mepolizumab*	20 (14)	14 (16)	5 (25)	1 (5)	0.396
*- Benralizumab*	23 (16)	16 (19)	3 (9)	4 (19)	0.428
*- Omalizumab*	9 (6)	5 (6)	2 (6)	2 (10)	0.820
*- Reslizumab*	1 (1)	1 (1)	0 (0)	0 (0)	0.729
*- Dupilumab*	40 (29)	31 (36)	4 (12)	5 (24)	0.031
*- Tezepelumab*	47 (34)	19 (22)	19 (58)	9 (43)	**<0.001**
***Smoking Status – n (%)***					0.205
-* Former*	62 (44)	34 (40)	19 (58)	9 (43)	
-* Never*	78 (56)	52 (61)	14 (42)	12 (57)	
***Packyears – Median (IQR)***	12 (5; 29)	10 (2.8; 24.8)	20 (5; 30)	10 (9; 20)	0.596
***Lung function – Median (IQR)***					
*FVC in % predicted*	85 (72; 96)	86 (73; 97)	84 (73; 93)	79 (65; 96)	0.389
*FEV1 in % predicted*	70 (55; 83)	71 (57; 84)	73 (50; 90)	59 (48; 76)	0.087
*RV in % predicted*	128 (102; 150)	126 (97; 147)	138 (106; 158)	131 (120; 162)	0.086
***Asthma Control Test – Median (IQR)***	16 (11-20)	19 (12-22)	14 (11-20)	13 (11-16)	**0.022**
***ExNO***	22 (11; 42)	18 (10; 44)	35 (12; 45)	22 (6; 33)	0.311
***Eosinophiles (absolute) – Median (IQR)***	0.07 (0.02; 0.21)	0.06 (0.02; 0.17)	0.14 (0.02; 0.3)	0.11 (0.02; 0.24)	0.553

Acronyms: BMI, body mass index; IQR, interquartile range. mAb, monoclonal antibody; FVC, Forced Vital Capacity; FEV_1_, Forced Expiratory Volume in one second; ExNO, Exhaled Nitric Oxide: RV, Residual Volume. Continuous variables are stated as median and interquartile ranges (IQR) and categorical variables are stated as n and percent (%). Statistically significant values in bold.

### Association between questionnaire results and treatment response

The PAM13 score showed no difference between response groups with a median (IQR) of 45 (42; 49) for all participants. Activation levels were distributed similarly among responders, partial responders and non-responders with no notable discrepancies in proportions. However, the A14 score differed significantly between responder and non-responder (p=0.013), partial-responder and non-responder (p=0.004 with no differences between responder and partial-responder. Partial responders present the highest median score of 53 (48; 55), compared to responders (51 [46; 54]) and non-responders (47 [43; 50]) (Table 2).

**Table 2:** Results of patient activation, adherence and knowledge.

	**All** **n=140**	**Responder** **n=86 (61%)**	**Partial responder** **n=33 (24%)**	**Non-responder** **n=21 (15%)**	**p-value**
***PAM13-Score – Median (IQR)***	45 (42; 49)	45 (43; 49)	45 (41; 49)	44 (39; 47)	0.184
*Slightly activated – n (%)*	1 (1)	1 (1)	0 (0)	0 (0)	0.179
*Moderately activated – n (%)*	5 (4)	0 (0)	3 (9)	2 (10)	
*Mostly activated – n (%)*	16 (12)	10 (12)	3 (9)	3 (14)	
*Strongly activated level – n (%)*	115 (84)	72 (87)	27 (82)	16 (76)	
***A14-Score – Median (IQR)***	50 (46; 54)	51 (46; 54)	53 (48; 55)	47 (43; 50)	0.004*
***A14-Adherent patients – n (%)***	75 (55%)	48 (58%)	22 (67%)	5 (25%)	**0.009**
*Own Regimen adaptations – Median (IQR)*	21 (18; 23)	21 (18; 23)	21 (16; 23)	18 (15; 21)	0.046*
*Practical obstacles in daily life – Median (IQR)*	19 (18; 20)	19 (18; 20)	20 (20; 20)	18 (17; 19)	<0.001*
*Negative attitude towards drugs – Median (IQR)*	8 (8; 8)	8 (8; 8)	8 (8; 8)	8 (6; 8)	0.002*
*Forgetfulness – Median (IQR)*	4 (3; 4)	4 (3; 4)	4 (4; 4)	3 (3; 4)	0.003*
***Asthma Knowledge– Median (IQR)***	45 (43; 48)	46 (43; 48)	46 (41; 48)	44 (42; 45)	0.014*
*Quality of life – Median (IQR)*	5 (4; 7)	6 (4; 8)	5 (3; 7)	5 (3; 7)	0.233
*Participant in disease management program – n (%)*	70 (53)	41 (49)	20 (67)	9 (47)	0.232
*Received asthma training – n (%)*	79 (57)	48 (57)	17 (52)	14 (67)	0.545
*Rehabilitation stay – n (%)*	74 (53)	42 (49)	19 (58)	13 (62)	0.501
*Training as part of rehabilitation – n (%)*	64 (56)	36 (50)	16 (59)	12 (75)	0.174
*Interested in further information – n (%)*	92 (67)	54 (64)	22 (67)	16 (76)	0.585

Acronyms: PAM13-Score, Patient Activation Measure (consisting of 13 questions); A14, Adherence to Treatment Questionnaire (consisting of 14 questions). Continuous variables are stated as median and interquartile ranges (IQR) and categorical variables are stated as n and percent (%). Statistically significant values in bold.

^*^ Pairwise Comparisons given with p values:

A14 score: responder–partial-responder: 1.000; responder–non-responder: **0.013**; partial-responder–non-responder: **0.004.**

Own Regimen adaptations: responder–partial-responder: 1.000; responder–non-responder: **0.047**; partial-responder–non-responder: 0.100.

Practical obstacles in daily life: responder–partial-responder: **0.030**; responder–non-responder: **0.018**; partial-responder–non-responder: **<0.001.**

Negative attitude towards drugs: responder–partial-responder: 1.000; responder–non-responder: **0.006**; partial-responder–non-responder: **0.002.**

Forgetfulness: responder–partial-responder: **0.020**; responder–non-responder: 0.592; partial-responder–non-responder: **0.006.**

Knowledge Score: responder–partial-responder: 1.000; responder–non-responder: **0.010**; partial-responder–non-responder: 0.091.

Regarding adherence-related behaviors, the median (IQR) A14 sub-score for own regimen adaptations was significantly lower in non-responders (18 [15; 21]) compared to responders (21 [18; 23]) and partial responders (21 [16; 23]), p=0.046. Non-responders also reported higher practical obstacles in daily life compared to responders and partial responders (p<0.001). Additionally, non-responders exhibited a more negative attitude towards drugs, with a median (IQR) score of 8 (6; 8) compared to responders (8 [8; 8]) and partial responders (8 [8; 8]), p=0.002. Forgetfulness was also more pronounced in non-responders with a median (IQR) score of 3 (3; 4), compared to responders (4 [3; 4]) and partial responders (4 [4; 4]), p=0.003. Asthma knowledge differed significantly among response groups (p=0.014), with partial responders and responders presenting the highest median score of 46 (41; 48) and 46 (43; 48) compared to non-responders (44 [42; 45]). Quality of life scores did not vary across response groups (p=0.233). All scores for the responder-groups are depicted in Fig. 1.

In ordinal logistic regression analyses, A14 score was not significantly associated with BARS-defined response category in univariate analysis (OR 0.96, 95% CI 0.904–1.015, p=0.145) or after adjustment for age, sex, smoking status, eosinophil levels and tezepelumab therapy (OR 0.94, 95% CI 0.880–1.021, p=0.115). Among the A14 subdomains, negative attitude towards drugs was associated with response category in univariate analysis (OR 0.51, 95% CI 0.028–0.919, p=0.025), but this association was attenuated after adjustment (OR 0.52, 95% CI 0.026–1.031, p=0.061). PAM13-D score was not significantly associated with response category in either univariate or adjusted analysis. Asthma knowledge was associated with response category in univariate analysis (OR 0.92, 95% CI 0.853–0.996, p=0.038) and remained significant after adjustment (OR 0.90, 95% CI 0.814–0.999, p=0.048). The effect size of this association was modest, and the confidence interval approached 1, indicating uncertainty regarding the magnitude of the association. Details are shown in Table 3. Assessment of the proportional odds assumption using the Brant test showed no relevant violation for the PAM13-D (omnibus p=0.10) and KISS models (omnibus p=0.12) based on non-significant omnibus tests. For the A14 model, the omnibus test was borderline significant (p=0.05) and the A14 score showed evidence of deviation from the proportional odds assumption; therefore, A14-related ordinal regression results were interpreted cautiously.

**Table 3:** Results of ordinal logistic regression analysis for outcome non-response.

	***Univariate regression***			Adjusted* regression		
	OR	95% Confidence interval	p-value	OR	95% Confidence interval	p-value
***A14-Score***	0.96	0.911; 1.017	0.182	0.94	0.880; 1.021	0.115
*Own Regimen adaptations*	0.94	0.875; 1.022	0.158	0.93	0.839; 1.032	0.174
*Practical obstacles in daily life*	0.95	0.794; 1.137	0.578	0.93	0.734; 1.173	0.531
*Negative attitude towards drugs*	0.51	0.028; 0.919	**0.025**	0.52	0.026; 1.031	0.061
*Forgetfulness*	1.29	0.739; 2.25	0.370	1.01	0.466; 2.174	0.986
***PAM13-D-Score***	0.95	0.892; 1.010	0.095	0.98	0.913; 1.059	0.655
***Asthma Knowledge***	0.921	0.853; 0.996	**0.038**	0.90	0.814; 0.999	**0.048**

Acronyms: A14, Adherence to Treatment Questionnaire (consisting of 14 questions). Statistically significant values in bold. *Models were adjusted for age, sex, smoking status, eosinophile levels and Tezepelumab.

## Discussion

Our study investigated the relationship between patient activation, adherence-related behaviors, asthma knowledge and treatment response in individuals with SEA undergoing monoclonal antibody treatment. Asthma knowledge remained associated with BARS-defined response category after adjustment, whereas PAM13-D and the overall A14 score were not independently associated with response. Among the A14 subdomains, a negative attitude towards drugs was associated with response category in univariate analysis, but this association was attenuated after adjustment. These findings suggest that patient-related factors, particularly disease-specific knowledge and medication attitudes, may be relevant for treatment response, although the cross-sectional design precludes causal interpretation. Further, mAb selection differed across response groups, with tezepelumab more frequently used in partial responders and non-responders. This finding should be interpreted cautiously and may reflect confounding by indication and treatment-selection effects rather than a causal effect of tezepelumab. Overall, given the cross-sectional design, these findings should be interpreted as associations and do not establish the direction of effect. It is also possible that patients with better treatment responses will become more engaged, more knowledgeable or more positive towards medication over time.

Assessment of adherence to inhaler medication by specific questionnaires has played a pivotal role in patient care for many years, as it is considered a key feature to improve disease control^27^. To our knowledge, there is no validated questionnaire specifically designed to determine treatment adherence for monoclonal antibody therapy. In a patient cohort treated with mepolizumab, adherence—defined as completing at least 9 out of 12 necessary drug administrations—was associated with improved disease outcomes comparable to our data^28^. Campisi et al. reported a positive effect of adherence (missed applications <10%) on ACT and exacerbation rate before and after treatment in omalizumab-treated patients^29^. These positive effects concerning exacerbations and emergency department visits were confirmed by large database studies^10,13^. In contrast, our adherence results are based on the A14 questionnaire, a cross-sectional patient-reported measure that includes subjective perceptions and does not directly capture medication administration data. According to our data, medication attitude may be relevant for treatment response, although the association was attenuated after adjustment and should therefore be interpreted cautiously. Of note, our approach also includes the use of inhaled drugs as a crucial step in asthma therapy and does not discriminate between different antibodies. Considering general medication use offers a plausible explanation for the difference in adherence rates observed between our patient cohort (55% adherence) and those in other studies focused solely on biologics therapy, which report adherence rates above 90%^28–30^. Nonetheless, adherence-related behaviors remain clinically relevant and may help identify patients who could benefit from additional education or adherence support. In addition to quantifying actual biologic administrations, rapid screening tools like the A14 questionnaire could be used to assess the patient’s individual adherence-related behaviors.

Our results showed higher asthma knowledge in responders and partial responders compared with non-responders, and asthma knowledge remained associated with BARS-defined response category after adjustment. To our knowledge, data on disease-specific knowledge and its relation to treatment response in patients receiving mAb therapy are lacking. Several cross-sectional studies confirmed a potential relationship between asthma knowledge and disease control, although both variables were assessed by different methods^31,32^. Of note, the BARS applied in our study consists of three aspects to assess treatment response instead of merely one in other studies^22^. Interestingly, improvement of asthma knowledge through online applications as well as respiratory nurse specialists resulted in significantly better disease control measured by ACT, with optimization of inhaler technique as a key aspect of these interventions^17,19^. However, it remains unclear whether our patient cohort benefited from these procedures, since a majority had already completed a disease management program, asthma-specific training, or pulmonary rehabilitation with training as part of rehabilitation. Thus, educational interventions addressing content specific to patients receiving monoclonal antibody therapy may be a valid tool to improve disease-specific knowledge and potentially improve treatment response. However, the effect size of the association between asthma knowledge and response category was modest and the adjusted p-value was borderline. Therefore, this finding should be considered hypothesis-generating and should not be interpreted as definitive evidence that improving asthma knowledge alone will improve biologic response.

Our findings suggest that response to biologic therapy in severe eosinophilic asthma is not solely determined by clinical characteristics but may be linked to modifiable patient-related factors. A more negative attitude towards medications was associated with non-response, but this association was attenuated after adjustment, indicating that behavioral barriers may contribute to suboptimal real-world effectiveness of monoclonal antibody therapy. These results support a practical, patient-centered approach in which adherence-related behaviors and medication attitudes are systematically assessed early during biologic treatment (e.g., at initiation and follow-up visits) to identify patients at risk for poor response. Targeted interventions, such as structured education addressing concerns about medications, individualized counseling, simplification of treatment routines, and practical support to overcome day-to-day obstacles, may help optimize therapeutic benefit before switching biologics or concluding treatment failure. Future prospective studies should test whether integrating attitude- and adherence-focused interventions into severe asthma care pathways improves response rates and clinical outcomes in patients receiving monoclonal antibodies.

Potential limitations of the study include its observational design, cross-sectional design, which precludes causal inference and does not allow conclusions regarding temporal directionality. In particular, lower adherence-related scores and less favorable medication attitudes may contribute to poorer treatment response, but poor response itself may also negatively affect patients’ attitudes and adherence behaviors. Longitudinal studies assessing adherence-related behaviors and asthma knowledge before and during biologic therapy are needed to clarify these relationships. Further, results of the questionnaires (adherence, patient activation) are based on subjective patient assessment. The questionnaire used to assess asthma knowledge is an adapted version and not fully validated with added items considered as clinically relevant by severe asthma specialists. Therefore, the association between KISS and BARS-defined response should be interpreted cautiously and requires confirmation using validated or prospectively evaluated knowledge instruments. Also, the A14 questionnaire may capture underlying adherence tendencies or attitudes relevant to biologic response, but it is not a direct measure of biologic dosing adherence and the proportional odds assumption was borderline for the A14 model; therefore, A14-related ordinal regression findings should be considered exploratory. Additionally, residual confounding cannot be excluded, as comorbidities, asthma duration, previous mAb use, mAb switching history, polypharmacy, educational level, and socioeconomic factors were not systematically assessed and may influence both asthma knowledge, adherence-related behaviors and treatment response, and should be included in future prospective studies. The generalizability of findings may be limited by the specific characteristics of the study population and is restricted to adults. Biologic selection differed across response groups (e.g., tezepelumab), which may reflect confounding by indication. Stratified analyses by biologic class, dosing interval, and prior biologic switching history were not feasible with the available data and should be addressed in future prospective studies. In addition, the proportional odds assumption was borderline for the A14 model; therefore, A14-related ordinal regression findings should be considered exploratory.

## Conclusion

Our study provides insights into patient-related factors associated with treatment response in individuals with severe asthma receiving mAb therapy. Asthma knowledge remained associated with BARS-defined response category after adjustment, whereas PAM13-D and overall A14 score were not independently associated with response. A more negative attitude towards medications was associated with response category in univariate analysis, but this association was attenuated after adjustment, suggesting that medication attitudes may still be clinically relevant but should be interpreted cautiously. These findings support a patient-centered approach in which asthma knowledge, adherence-related behaviors and medication attitudes are considered during biologic treatment. Prospective studies are needed to determine whether systematic assessment and targeted educational or adherence-support interventions improve response rates and clinical outcomes in real-world asthma care.

## Supplementary Information

Supplementary Information 1.

Supplementary Information 2.

## Data Availability

Anonymized data will be provided by corresponding author on reasonable request.
