# Supplementary material for: Relationship between knowledge, adherence-related behaviors and self-management with response to monoclonal antibody therapy in patients with severe asthma
Source: Sci Rep. 2026 Jul 31;16:23716. doi: 10.1038/s41598-026-64363-5 (PMC13427718; doi:10.1038/s41598-026-64363-5)
Supplement: Supplementary file 1 — Supplementary Information 1. [file 41598_2026_64363_MOESM1_ESM.docx]

STROBE Statement—checklist of items that should be included in reports of observational studies

|  | Item No. | Recommendation | Page  No. | Relevant text from manuscript |
| --- | --- | --- | --- | --- |
| **Title and abstract** | 1 | (*a*) Indicate the study’s design with a commonly used term in the title or the abstract |  |  |
|  |  | (*b*) Provide in the abstract an informative and balanced summary of what was done and what was found | 2 | Participants diagnosed with SEA and undergoing mAb therapy for 6-9 months were recruited from three German university outpatient clinics. Patients were categorized into responders, partial responders and non-responders using the Biologics Asthma Response Score (BARS). Data collection focused on demographic details, lung function, medication history, asthma-knowledge, patient activation (PAM13-D), and adherence (A14 questionnaire). Statistical analyses assessed the impact of adherence and knowledge on treatment outcomes. |
| Introduction | | | |  |
| Background/rationale | 2 | Explain the scientific background and rationale for the investigation being reported | 3 | Although several monoclonal antibodies have been approved for use in severe asthma, there remains a subset of patients who do not achieve full therapeutic benefit from these biologic agents. Adherence, patient activation or asthma knowledge might play a role. |
| Objectives | 3 | State specific objectives, including any prespecified hypotheses | 4 | This study aims to elucidate the interplay between patient activation, adherence-related behaviors, asthma knowledge and response to monoclonal antibody treatment in a large cohort of patients with mAb-therapy for severe asthma |
| Methods | | | |  |
| Study design | 4 | Present key elements of study design early in the paper | 4 | Patients diagnosed with SEA and mAb therapy for at least 6 to 9 months were recruited for an observational cohort study between November 2022 until November 2023 from three German university outpatient clinics for severe asthma |
| Setting | 5 | Describe the setting, locations, and relevant dates, including periods of recruitment, exposure, follow-up, and data collection | 4 | Patients diagnosed with SEA and mAb therapy for at least 6 to 9 months were recruited for an observational cohort study between November 2022 until November 2023 from three German university outpatient clinics for severe asthma: Ludwig-Maximilian-Universität in Munich, Thoraxklinik Heidelberg and Medizinische Hochschule Hannover |
| Participants | 6 | (*a*) *Cohort study*—Give the eligibility criteria, and the sources and methods of selection of participants. Describe methods of follow-up  *Case-control study*—Give the eligibility criteria, and the sources and methods of case ascertainment and control selection. Give the rationale for the choice of cases and controls  *Cross-sectional study*—Give the eligibility criteria, and the sources and methods of selection of participants | 4 | Patients diagnosed with SEA and mAb therapy for at least 6 to 9 months were recruited |
|  |  | (*b*) *Cohort study*—For matched studies, give matching criteria and number of exposed and unexposed  *Case-control study*—For matched studies, give matching criteria and the number of controls per case | - | - |
| Variables | 7 | Clearly define all outcomes, exposures, predictors, potential confounders, and effect modifiers. Give diagnostic criteria, if applicable | 4 | See paragraph “data collection” |
| Data sources/ measurement | 8* | For each variable of interest, give sources of data and details of methods of assessment (measurement). Describe comparability of assessment methods if there is more than one group | 4 | See paragraph “data collection” |
| Bias | 9 | Describe any efforts to address potential sources of bias | 4 | There were no specific efforts taken to address for bias other then we tried to recruit as much patients as possible for this study to minimize bias. |
| Study size | 10 | Explain how the study size was arrived at | 4 | All patients eligible were asked to participate in this study, there is no formal sample size calculation. |

Continued on next page

| Quantitative variables | 11 | Explain how quantitative variables were handled in the analyses. If applicable, describe which groupings were chosen and why | 5 | Statistical analysis was conducted to assess differences in patient activation, adherence-related behaviors, and other variables across responder, partial responder, and non-responder groups. Descriptive statistics such as median and interquartile range (IQR) or mean and standard deviation (SD) were used to summarize continuous variables, while categorical variables were shown using frequencies and percentages. Group differences were analyzed using appropriate statistical tests, including Kruskal-Wallis test for continuous variables and chi-square test for categorical variables. |
| --- | --- | --- | --- | --- |
| Statistical methods | 12 | (*a*) Describe all statistical methods, including those used to control for confounding | 5 | IBM SPSS Statistics version 29 and R-Studio using R 4.3.3 were used to analyze the data. Statistical analysis was conducted to assess differences in patient activation, adherence-related behaviors, and other variables across responder, partial responder, and non-responder groups. Descriptive statistics such as median and interquartile range (IQR) or mean and standard deviation (SD) were used to summarize continuous variables, while categorical variables were shown using frequencies and percentages. Group differences were analyzed using appropriate statistical tests, including Kruskal-Wallis test for continuous variables and chi-square test for categorical variables. Simple and multiple ordinal regression were calculated to evaluate the impact of A14 score and KISS on non-response category of BARS. Variables with p-values <0.1 were included in multiple regression model. Significance was set at p < 0.05. |
|  |  | (*b*) Describe any methods used to examine subgroups and interactions | 5 | See above |
|  |  | (*c*) Explain how missing data were addressed | - | Not applicable |
|  |  | (*d*) *Cohort study*—If applicable, explain how loss to follow-up was addressed  *Case-control study*—If applicable, explain how matching of cases and controls was addressed  *Cross-sectional study*—If applicable, describe analytical methods taking account of sampling strategy | - | Not applicable |
|  |  | (*e*) Describe any sensitivity analyses | - | Not applicable |
| Results | | | | |
| Participants | 13* | (a) Report numbers of individuals at each stage of study—eg numbers potentially eligible, examined for eligibility, confirmed eligible, included in the study, completing follow-up, and analysed | 5 | 140 Patients could be included in the study of which 54% were female with a median age of 57 (51; 64) years. |
|  |  | (b) Give reasons for non-participation at each stage | - | All patients asked participated |
|  |  | (c) Consider use of a flow diagram | - | Flowchart gives no additional information, therefore we decided not to include any |
| Descriptive data | 14* | (a) Give characteristics of study participants (eg demographic, clinical, social) and information on exposures and potential confounders | 5 and Table 1 | 140 Patients could be included in the study of which 54% were female with a median age of 57 (51; 64) years. |
|  |  | (b) Indicate number of participants with missing data for each variable of interest | - | Not applicable |
|  |  | (c) *Cohort study*—Summarise follow-up time (eg, average and total amount) | 4 | Patients diagnosed with SEA and mAb therapy for at least 6 to 9 months were recruited |
| Outcome data | 15* | *Cohort study*—Report numbers of outcome events or summary measures over time | *5* | 140 Patients could be included in the study of which 54% were female with a median age of 57 (51; 64) years. Participants were categorized into responder (n=86, 61%), partial responder (n=33, 24%) and non-responder (n=21, 15%) using the BAR score. |
|  |  | *Case-control study—*Report numbers in each exposure category, or summary measures of exposure |  |  |
|  |  | *Cross-sectional study—*Report numbers of outcome events or summary measures |  |  |
| Main results | 16 | (*a*) Give unadjusted estimates and, if applicable, confounder-adjusted estimates and their precision (eg, 95% confidence interval). Make clear which confounders were adjusted for and why they were included | 6 | Among the predictors examined in ordinal logistic regression analysis, a negative attitude towards drugs showed a statistically significant association with non-response in both the univariate (p = 0.021) and multivariate (p = 0.020) regression analyses. Asthma knowledge did not show statistically significant associations with non-response, albeit showing a trend with p=0.051. Details are shown in Table 3. |
|  |  | (*b*) Report category boundaries when continuous variables were categorized |  |  |
|  |  | (*c*) If relevant, consider translating estimates of relative risk into absolute risk for a meaningful time period |  |  |

Continued on next page

| Other analyses | 17 | Report other analyses done—eg analyses of subgroups and interactions, and sensitivity analyses |  |  |
| --- | --- | --- | --- | --- |
| Discussion | | | | |
| Key results | 18 | Summarise key results with reference to study objectives | 7 and 8 | Our study investigated the relationship between patient activation, adherence-related behaviors, asthma knowledge and treatment response in individuals with SEA undergoing monoclonal antibody treatment. We found that greater adherence to asthma medication regimens were positively correlated with improved treatment response whereas individuals with a more negative attitude towards drugs are more likely to exhibit non-response to treatment. Our study identified a potential role for asthma knowledge in treatment response. Participants with a better understanding of asthma and its management demonstrated more favorable treatment response. |
| Limitations | 19 | Discuss limitations of the study, taking into account sources of potential bias or imprecision. Discuss both direction and magnitude of any potential bias | 8 | Potential limitations of the study included its observational design which precludes establishment of causal relationships and the possibility of selection bias inherent in the recruitment process. Further, the questionnaire used to assess asthma knowledge is an adapted version and not fully validated. Additionally, the generalizability of findings may be limited by the specific characteristics of the study population. |
| Interpretation | 20 | Give a cautious overall interpretation of results considering objectives, limitations, multiplicity of analyses, results from similar studies, and other relevant evidence | 8 | In conclusion, our study provides valuable insights into the factors influencing treatment response in individuals with severe asthma. By elucidating the relationships between patient activation, adherence-related behaviors, asthma knowledge and treatment outcomes, our findings highlight the importance of adopting a patient-centered approach to asthma care. Moving forward, interventions aimed at enhancing patient activation, promoting medication adherence, and providing comprehensive asthma education may hold promise in improving treatment outcomes and enhancing the quality of life for individuals living with severe asthma. |
| Generalisability | 21 | Discuss the generalisability (external validity) of the study results | 8 | Further research is warranted to explore the effectiveness of these interventions in real-world clinical settings and to identify additional factors influencing treatment response in this complex patient population. |
| Other information | |  | | |
| Funding | 22 | Give the source of funding and the role of the funders for the present study and, if applicable, for the -original study on which the present article is based |  | Not applicable |

*Give information separately for cases and controls in case-control studies and, if applicable, for exposed and unexposed groups in cohort and cross-sectional studies.

**Note:** An Explanation and Elaboration article discusses each checklist item and gives methodological background and published examples of transparent reporting. The STROBE checklist is best used in conjunction with this article (freely available on the Web sites of PLoS Medicine at http://www.plosmedicine.org/, Annals of Internal Medicine at http://www.annals.org/, and Epidemiology at http://www.epidem.com/). Information on the STROBE Initiative is available at www.strobe-statement.org.
