# Supplementary material for: Relationship between knowledge, adherence-related behaviors and self-management with response to monoclonal antibody therapy in patients with severe asthma
Source: Sci Rep. 2026 Jul 31;16:23716. doi: 10.1038/s41598-026-64363-5 (PMC13427718; doi:10.1038/s41598-026-64363-5)
Supplement: Supplementary file 2 — Supplementary Information 2. [file 41598_2026_64363_MOESM2_ESM.docx]

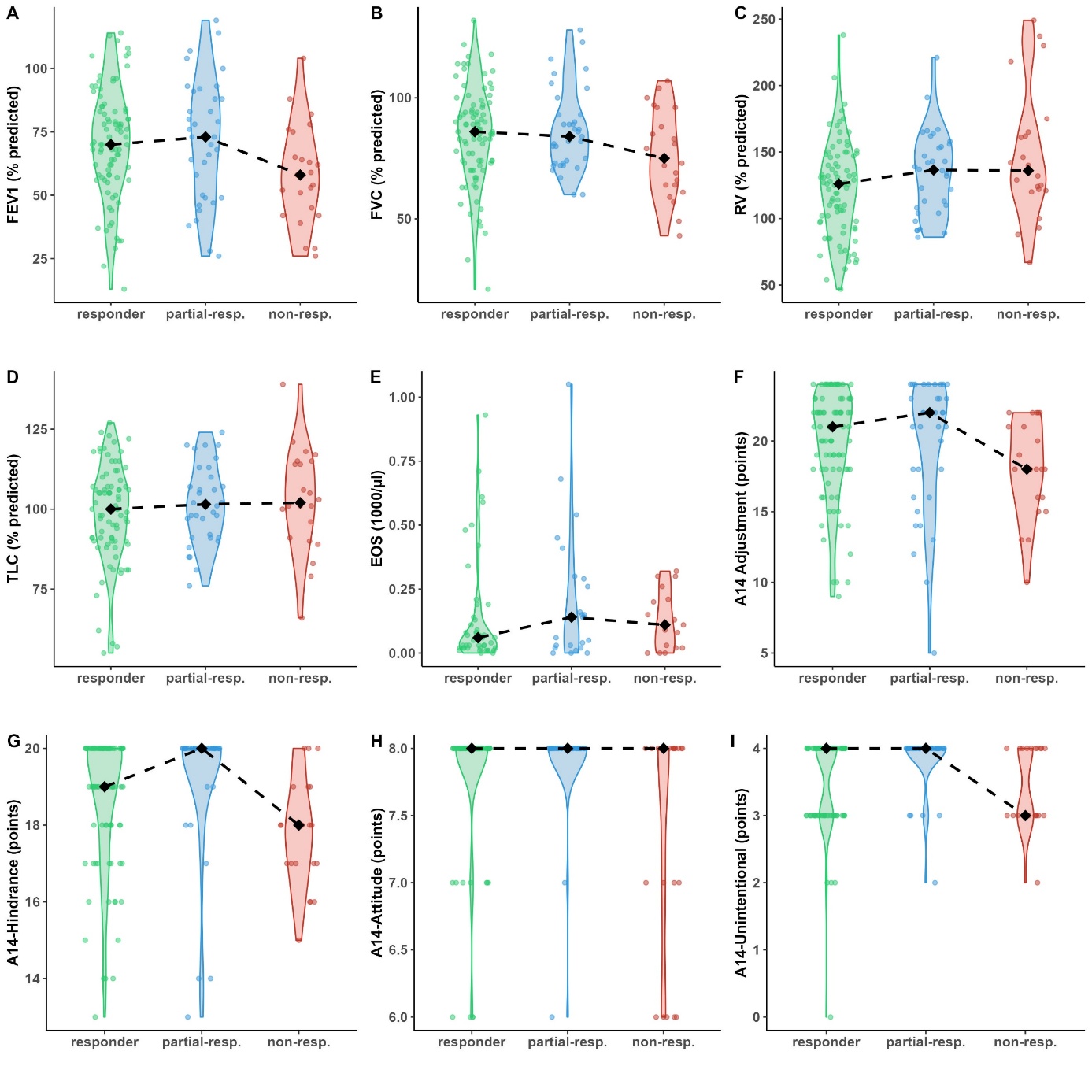


**Figure S1:** Comparison between responder, partial-responder and non-responder for (A) Forced expiratory volume in one second (FEV1, % predicted), (B) Forced vital capacity (FVC, % predicted), (C) Residual volume (RV, % predicted), (D) Total lung capacity (TLC, % predicted), (E) Eosinophile count (EOS, in 1000/µl), (F) A14, Adherence to Treatment Questionnaire, Sub score for adjustment (A14-adjustment), (G) Adherence to Treatment Questionnaire, Sub score for hindrance (A14-hindrance), (H) Adherence to Treatment Questionnaire, Sub score for attitude (A14-attitude), (I) Adherence to Treatment Questionnaire, Sub score for unintentional non-adherence (A14-unintentional). Data are presented as violin plots with Median and individual data points.

Table S1 – Patient demographics, Lung functions and questionnaire results by adherence category

|  | Adherent n=78 (56%) | Non-Adherent n=62 (44%) | p-value |
| --- | --- | --- | --- |
| Female sex – n (%) | 49 (65) | 27 (35) | **0.023** |
| Age – Median (IQR) | 58 (52; 67) | 56 (48; 62) | **0.049** |
| BMI – Median (IQR) | 27 (24; 32) | 28 (24; 34) | 0.215 |
| **mAb-Therapy – n (%)** |  |  |  |
| - Mepolizumab | 12 (16) | 8 (13) | 0.677 |
| - Benralizumab | 3 (9) | 4 (19) | 0.428 |
| - Omalizumab | 6 (8) | 3 (5) | 0.494 |
| - Reslizumab | 1 (1) | 0 (0) | 0.371 |
| - Dupilumab | 4 (12) | 5 (24) | 0.031 |
| - Tezepelumab | 23 (29) | 24 (39) | 0.251 |
| **Smoking Status – n (%)** |  |  | 0.618 |
| - Former | 36 (58) | 26 (42) |  |
| - Never | 42 (54) | 36 (46) |  |
| **Lung function – Median (IQR)** |  |  |  |
| FVC in % predicted | 88 (74; 97) | 81 (70; 91) | **0.022** |
| FEV1 in % predicted | 72 (57; 88) | 66 (50; 80) | **0.034** |
| RV in % predicted | 131 (99; 149) | 127 (106; 150) | 0.391 |
| ExNO | 35 (12; 45) | 22 (6; 33) | 0.311 |
| Eosinophiles (absolute) – Median (IQR) | 0.08 (0.02; 0.26) | 0.07 (0.02; 0.2) | 0.849 |
| PAM13-Score – Median (IQR) | 46 (43; 50) | 44 (40; 47) | **0.006** |
| Asthma Knowledge– Median (IQR) | 45 (42; 48) | 45 (43; 48) | 0.859 |

Acronyms: BMI, body mass index; IQR, interquartile range. mAb, monoclonal antibody; FVC, Forced Vital Capacity; FEV1, Forced Expiratory Volume in one second; ExNO, Exhaled Nitric Oxide: RV, Residual Volume. Continuous variables are stated as median and interquartile ranges (IQR) and categorical variables are stated as n and percent (%). Statistically significant values in bold.

Table S2 – Demographic, clinical and questionnaire-based characteristics according to biologic therapy

|  | **Mepolizumab (N=20)** | **Benralizumab (N=23)** | **Omalizumab/ Reslizumab (N=10)** | **Dupilumab (N=40)** | **Tezepelumab (N=47)** | **p value** |
| --- | --- | --- | --- | --- | --- | --- |
| Female sex – n (%) | 12 (60%) | 15 (65%) | 8 (80%) | 22 (55%) | 19 (40%) | 0.104 |
| Age – Median (IQR) | 58.0 (47.5, 66.2) | 62.0 (58.0, 68.5) | 55.5 (48.0, 56.8) | 59.0 (50.0, 64.2) | 55.0 (50.5, 60.0) | 0.030 |
| BMI – Median (IQR) | 26.2 (23.7, 29.9) | 25.9 (23.8, 34.6) | 23.2 (21.9, 26.4) | 27.8 (23.6, 31.9) | 29.0 (25.5, 32.9) | 0.104 |
| **Smoking Status – n (%)** |  |  |  |  |  | 0.320 |
| - Former | 11 (55%) | 15 (65%) | 5 (50%) | 26 (65%) | 21 (45%) |  |
| - Never | 9 (45%) | 8 (35%) | 5 (50%) | 14 (35%) | 26 (55%) |  |
| **Packyears – Median (IQR)** | 30.0 (5.0, 40.0) | 10.0 (2.0, 11.2) | 22.0 (10.8, 49.5) | 9.5 (2.8, 20.8) | 15.0 (7.0, 26.0) | 0.508 |
| **Lung function – Median (IQR)** |  |  |  |  |  |  |
| FEV1 in % predicted | 69.5 (54.2, 83.5) | 73.0 (66.0, 82.0) | 73.0 (60.5, 81.2) | 73.0 (55.2, 85.2) | 64.0 (51.0, 82.5) | 0.676 |
| FVC in % predicted | 82.5 (71.2, 93.2) | 89.0 (82.0, 97.0) | 81.0 (68.2, 94.2) | 84.0 (72.8, 104.8) | 83.0 (72.5, 92.5) | 0.751 |
| RV in % predicted | 127.0 (109.0, 147.2) | 128.0 (92.5, 144.5) | 109.0 (98.0, 110.0) | 122.0 (93.0, 147.0) | 136.5 (113.5, 157.5) | 0.115 |
| **Asthma Control Test – Median (IQR)** | 17.0 (9.8, 19.2) | 21.0 (17.0, 24.0) | 16.0 (10.5, 22.2) | 19.0 (12.0, 21.2) | 11.0 (10.0, 16.0) | **< 0.001** |
| **ExNO** | 58.0 (26.0, 71.0) | 30.5 (24.0, 43.1) | 12.5 (10.5, 17.0) | 17.5 (9.9, 35.5) | 16.0 (8.7, 36.0) | **0.002** |
| **Eosinophiles (absolute) – Median (IQR)** | 0.0 (0.0, 0.4) | 0.0 (0.0, 0.0) | 0.1 (0.0, 0.1) | 0.1 (0.1, 0.3) | 0.1 (0.0, 0.2) | 0.090 |
| **A14-Score – Median (IQR)** | 51.5 (45.8, 54.0) | 51.0 (43.0, 53.5) | 52.0 (47.8, 53.8) | 52.0 (48.0, 55.0) | 49.0 (45.0, 53.5) | 0.581 |
| Own Regimen adaptations – Median (IQR) | 20.5 (15.8, 23.0) | 21.0 (14.0, 22.0) | 20.0 (18.0, 22.0) | 22.0 (19.0, 23.0) | 20.0 (17.5, 22.0) | 0.301 |
| Practical obstacles in daily life – Median (IQR) | 20.0 (16.8, 20.0) | 19.0 (18.0, 20.0) | 20.0 (18.0, 20.0) | 19.0 (18.0, 20.0) | 19.0 (17.5, 20.0) | 0.968 |
| Negative attitude towards drugs – Median (IQR) | 8.0 (8.0, 8.0) | 8.0 (7.0, 8.0) | 8.0 (8.0, 8.0) | 8.0 (8.0, 8.0) | 8.0 (8.0, 8.0) | 0.370 |
| Forgetfulness – Median (IQR) | 4.0 (3.0, 4.0) | 4.0 (3.0, 4.0) | 4.0 (3.0, 4.0) | 4.0 (3.0, 4.0) | 4.0 (3.0, 4.0) | 0.769 |
| **PAM13-Score – Median (IQR)** | 46.0 (43.0, 51.0) | 45.0 (43.0, 48.0) | 43.5 (40.0, 46.5) | 46.5 (43.0, 50.0) | 43.5 (39.0, 46.0) | **0.048** |
| **Asthma Knowledge– Median (IQR** | 42.0 (39.0, 48.0) | 46.0 (43.0, 48.0) | 46.0 (44.2, 48.0) | 45.0 (43.0, 47.0) | 45.0 (43.0, 48.0) | 0.366 |

Acronyms: ACT, Asthma Control Test; BMI, body mass index; ExNO, exhaled nitric oxide; FEV1, forced expiratory volume in one second; FVC, forced vital capacity; IQR, interquartile range; PAM13-D, Patient Activation Measure 13, German version; RV, residual volume. Continuous variables are stated as median and interquartile range (IQR), and categorical variables are stated as n and percent (%). Statistically significant values are shown in bold.
